# The Surgery of Gummatous Growths of the Nasal Cavities

**Published:** 1888-11

**Authors:** A. G. Hobbs

**Affiliations:** Professor of Opthalmology, Otology, Rhino-Laryngology in Southern Medical College, Atlanta, Ga.


### Southern Medical Record.

A MONTHLY JOURNAL OF PRACTICAL MEDICINE.

☞All Communications must be addressed to R. C. WORD, M. D., Managing Editor

"*Quicquid Præcipies Esto Brevis.*"

VOL. XVIII. ATLANTA, GA., NOVEMBER, 1888. No. 11.

### ORIGINAL AND SELECTED ARTICLES.

### THE SURGERY OF GUMMATOUS GROWTHS OF THE NASAL CAVITIES.*

BY A. G. HOBBS, M. D.
Professor of Opthalmoloy, Otology, Rhino-Laryngology in Southern Medical College
Atlanta, Ga.

I will report the following cases of gummatous growth occurring in the nasal cavities: 1. Because of their comparative rarity and 2. Because the treatment of these four cases has not been according to the conventional mode, if I may judge by the text books and journals as well as by my own early teachings. I believe it has been the general rule not to resort to surgical procedures to reduce gummatous tumors when found in the nasal cavities from fear of causing destructive ulcerations. The following four cases are all that have come under my observation during the past three years, and the treatment resorted to in them gave me entirely satisfactory results; this cannot be said of the few cases I saw and attempted to treat previous to this time.

CASE 1. In June of 1885, Dr. Robinson, of Atlanta, brought a patient to me to be treated for nasal stenosis with the following history: A young married woman had had syphilis for three years with the usual treatment, no symptoms having shown themselves during the past year. Three months before I saw her, she began to feel a "stuffiness" about her nose, in one nostril particularly, which gradually increased until she breathed entirely through

*Read before the American Rhionological Association at Cincinnati, Sept. 14, 1888

her mouth. An anterior examination revealed a purplish red growth which filled the right nasal space completely and pressed itself into the left cavity till the stenosis was almost, if not quite, complete. I could not ascertain much by a posterior examination on account of an entremely irritable throat and a rebellious tongue. From the history of the case and the appearance of the growth its gummatous nature was suspected, hence it scarcely occurred to me at this time, to attempt its removal by any surgical means for fear of preducing a destructive ulceration.

She was placed upon increased doses of potassium iodide together with biniodide of mercury. This treatment was continued four weeks when she returned unimproved, indeed she was in a still worse condition from being compelled to breathe entirely through her mouth. The tumor presented no changes—it had at least not decreased in size. I then determined to undertake the immediate removal of the growth, explaining to her the possible results. I cocainized the nasal passages with a 5% spray as thoroughly as was possible, when I attempted to introduce a Blake's snare, but found it impossible to engage the tumor in the loup. A cutting spoon was then introduced and several large masses of the growth cut away. I was supprised at the small hemorrhage which did not amount to more than a drachm or two. When the flow had ceased she could breathe through the left nostril. Four more sittings at intervals of three days were necessary to remove the entire growth. The attachment was by a broad pedicle at the posterior part of the septum which I seared with the galvano-cautery when the last particle of the growth that could be seen had been extracted. Considerable more hemorrhage, possibly two ounces altogether, followed the succeeding sittings. The patient was then furnished with a spray apparatus. No ulceration followed, and Dr. Robinson reported six months afterwards that there had been no recurrence, and that she had regained her health completely. She was kept on anti-specific treatment during the six months.

CASE 2. In November of 1885, Dr. Abry referred to me a gentleman aged 44, who, he thought, was suffering from hypertrophic nasal catarrh, stating at the same time that he had treated him two years previously for syphilis. I treated the hypertrophy daily for about a month, when it had responded so promptly to the treatment that he considered himself sufficiently recovered to leave for California. He did not return for more than four months when he presented himself remarking that his nose was almost completely closed, entirely so when lying down. It gave him such urgent discomfort that he insisted upon my doing something, anything to relieve him. An anterior examination revealed a purplish red growth, one on each side, attached at the posterior part of the septum; these growth had not existed four months previously—he thought his nose had been closing up, as he expressed it, about two months. After a thorough use of cocaine, I again attempted to use the snare—using Jarvis' this time—and with more success than in the first case. I succeeded in removing a large piece from each nostril at one sitting; the screw was turned very slowly and the hemorrhage was inconsiderable. Three days afterwards I pinched off and scraped away the last remaining trace of the growths and seared the stumps with the cautery. No ulceration followed, but it required a two months use of the spray to heal the resulting catarrh. He was then dismissed with a succus alterans and iodide alterative to be continued six months. There has been no recurrence to this date.

CASE 3. A young mulatto woman of very nervous temperament and the usual scrofulous diathesis, was brought to me by her family physician, for nasal stenosis. There was no distinct syphilitic history nor any symptoms other than some copper colored skin blotches on her neck and shoulders that were somewhat suspicious. Her physician had suspected syphilis, but had not given her any anti-specific treatment. The nasal tumor that occupied the right nasal space and insinuated itself into the left presented a very similar appearance to case first. In consequence of being unable to sleep with her mouth closed, she arose in the morning after a night of snoring with a dry, parched mouth and throat and a dull, heavy headache. She had first noticed the closing of the right nostril about four months previous to her first visit to me in March, 1887. The growth presented all the characteristics of gummatous tumor, notwithstanding the absence of specific history and the paucity of specific symptoms. After thoroughly cocainizing I removed the greater part of the growth with cutting edged forceps; considerable hemorrhage followed on account of the use of the forceps perhaps, but I had been unable to engage the loup of the snare, so completely was the space filled and so broad was the attachment. Three days afterwards I removed all the remaining portions of the tumor with t e curette and forceps and seared the broad base. She was provided with a vaseline spray and instructed to return if she had any more trouble.

Two weeks afterwards she returned complaining of soreness and "stuffiness" in the nose. I found upon examination, a deep ulcer occupying the seat of the tumor's attachment. It caused me considerable trouble during the next two weeks. I treated it with applications of nitrate of silver full strength on a cotton probe; some bony necrosis followed, but healing had taken place in the course of three weeks and the resulting cicatrix was not an ugly one or an inconvenient one. She was advised to continue a specific alterative of potassium iodide and succus alterans for six months. When I heard from her some weeks afterwards, she had gained eight pounds in weight and her health was completely restored.

CASE 4. Was an old Scotchman, aged 66. If he had ever had syphilis he did not know it by that name; said he had an ulcerated sore throat with a skin eruption in Scotland twenty years ago, which lasted him many months, and for which he was treated six or eight months. An ugly cicatrix covered his pharynx and the velum was adherent in more than half of its extent to the posterior wall of the pharynx. He came to me to be treated for deafness and tinnitus in the left ear.

Upon examining his nasal cavities anteriorly, a growth was found in the left nasal space which presented all the characteristic appearances of a gummatous tumor. As the specific history and symptoms were meagre, I pinched off a small piece for microscopic examination. It presented all the peculiarities, under the microscope, of the preceding cases, and corresponded with a Heitzman specimen of a gummatous growth in the nasal cavities, in my possession.

My first attempt at extracting it produced great pain and decided hemorrhage, notwithstanding cocaine was applied as thoroughly as was possible. I succeeded, however, in pinching away about half of it at the first sitting. I concluded the day afterwards when he next returned to cocainize and try the application of chromic acid. I repeated the application of chromic acid, fused upon the end of a platinum wire, daily for ten days, wheu the entire mass had been extracted principally in small pieces, much less pain and no hemorrhrge was produced by this method. The middle ear catarrh which had been caused by the pressure of the tumor at the eustachian orifice, became very much worse after a few day's application of the chromic acid to the growth, but it finally passed away as the stenasis lessened, without producing a rupture of the drum membrane.

The resulting nasal catarrh was treated with vaseline sprays for six weeks, when he was dismissed. He was not placed upon a specific treatment, but was advised to return to me at once when he discovered any nasal trouble. I have heard nothing further from him since his dismissal in May of this year.

My deductions from these cases occurring in my practice during the last three years are that: 1. Surgery is not only admissible in most cases of this character, but in many instances it becomes urgently necessary. 2. The fear of producing destructive, at least uncontrollable, ulcerations has not a sufficient clinical foundation. 3. Alterative anti-syphilitic treatment is too slow to be depended upon alone when the stenosis is producing so many reflex symptoms, such as asthma, coughs, sleeplessness, headache, loss of weight, dry parched throat, etc. 4. The loss of blood from instrumental means is not dangerous nor even alarming, and while the pain is considerable, as is the case in removing most nasal growths, it can be greatly mitigated by cocaine.

